# In depth analysis of the *Sox4* gene locus that consists of sense and natural antisense transcripts

**DOI:** 10.1016/j.dib.2016.01.045

**Published:** 2016-02-17

**Authors:** King-Hwa Ling, Peter J. Brautigan, Sarah Moore, Rachel Fraser, Melody Pui-Yee Leong, Jia-Wen Leong, Shahidee Zainal Abidin, Han-Chung Lee, Pike-See Cheah, Joy M. Raison, Milena Babic, Young Kyung Lee, Tasman Daish, Deidre M. Mattiske, Jeffrey R. Mann, David L. Adelson, Paul Q. Thomas, Christopher N. Hahn, Hamish S. Scott

**Affiliations:** a Department of Molecular Pathology, The Institute of Medical and Veterinary Science and The Hanson Institute, P.O. Box 14 Rundle Mall Post Office, Adelaide, SA 5000, Australia; b School of Medicine, Faculty of Health Sciences, University of Adelaide, Adelaide, SA 5005, Australia; c NeuroBiology & Genetics Group, Genetics and Regenerative Medicine Research Centre, Faculty of Medicine and Health Sciences, Universiti Putra Malaysia, 43400 UPM Serdang, Selangor DE, Malaysia; d School of Biological Sciences, Faculty of Sciences, University of Adelaide, Adelaide, SA 5005, Australia; e Department of Human Anatomy, Faculty of Medicine and Health Sciences, Universiti Putra Malaysia, UPM, 43400 Serdang, Selangor DE, Malaysia; ^f^Theme of Laboratory and Community Genetics, Murdoch Childrens Research Institute, Royal Children׳s Hospital, Flemington Road, Parkville, VIC 3052, Australia; ^g^Biomedicine Discovery Institute, Monash University, VIC 3800, Australia

**Keywords:** Endogenous siRNA, Brain development, Natural antisense transcripts

## Abstract

SRY (Sex Determining Region Y)-Box 4 or *Sox4* is an important regulator of the pan-neuronal gene expression during post-mitotic cell differentiation within the mammalian brain. *Sox4* gene locus has been previously characterized with multiple sense and overlapping natural antisense transcripts [1], [2]. Here we provide accompanying data on various analyses performed and described in Ling et al. [2]. The data include a detail description of various features found at *Sox4* gene locus, additional experimental data derived from RNA-Fluorescence *in situ* Hybridization (RNA-FISH), Western blotting, strand-specific reverse-transcription quantitative polymerase chain reaction (RT-qPCR), gain-of-function and *in situ* hybridization (ISH) experiments. All the additional data provided here support the existence of an endogenous small interfering- or PIWI interacting-like small RNA known as *Sox4*_sir3, which origin was found within the overlapping region consisting of a sense and a natural antisense transcript known as *Sox4ot1*.

## Specifications Table

**Table:** 

Subject area	*Biology.*
More specific subject area	*RNA Biology or Neurogenetics.*
Type of data	*Genbank file, table, bar charts, micrographs, MOV files and statistical analysis*
How data was acquired	*C57BL/6 mice, Artemis visualization tool, LightCycler® 480 System, Zeiss Axioplan 2 Imaging upright microscope with Axiovision software, ImageJ software, GraphPad Prism®.*
Data format	*Filtered and analyzed.*
Experimental factors	*Real-time/Reverse-transcription quantitative polymerase chain reaction (RT-qPCR), Western and Southern blotting analyses, rapid amplification of cDNA Ends, RNA-Fluorescence in situ Hybridization on different brain cells, LNA-ISH of the developing embryo/adult brain and overexpression analysis.*
Experimental features	*Multi-approach molecular and cellular characterization of Sox4 gene locus in experimental house mouse model (Mus musculus).*
Data source location	*Universiti Putra Malaysia, Selangor, Malaysia and University of Adelaide, South Australia, Australia.*
Data accessibility	*The data is available with this article.*

## Value of the data

• The data describes the derivation of an endogenous small RNA via double-stranded RNA template in the mouse. This is a rare event within the mammalian genome but is common in the plant.
• The data provides a modified method for brain cell fixation and immobilisation on glass slides for effective RNA-FISH analysis.
• Comparison of two different *Sox4* natural antisense transcripts, known as *Sox4ot1* and *Sox4ot2* in the production of *Sox4*_sir3 *in vitro*.
• Compilation of all the information within the *Sox4* gene locus allows clear, concise and easy visualisation of various features defined in the region by using Artemis software.

## 1 Data, experimental design, materials and methods

### 1.1 Genomics mapping of various features within Sox4 gene locus

**Fig. 1:**
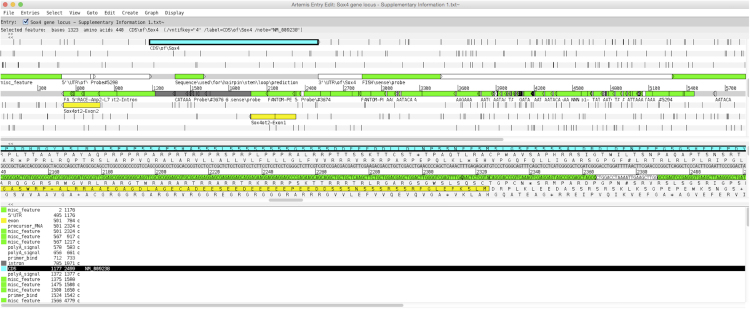
A snapshot of *Sox4* gene locus. The *Sox4* gene locus visualized using Artemis Genome Browser and Annotation Tool.

The data reported here consists of information related to the *Sox4* gene locus. The *Sox4* gene locus is featured by multiple overlapping sense and natural antisense transcripts (NATs) [1], [2]. Various efforts such as Serial Analysis of Gene Expression (SAGE) [1] and Rapid Amplification of cDNA Ends (RACE) in combination with strand specific Southern blotting analysis [2] were performed to characterize the locus. *In silico* data mining and mapping were also carried out to enrich the features within the locus and the detailed information is summarized in a GenBank file format as Supplementary GenBank File. A snapshot of the annotated *Sox4* gene locus visualized using Artemis Genome Browser and Annotation Tool [3] is illustrated in Fig. 1. Information embedded within Supplementary GenBank File includes the sequences and loci for predicted NATs based on RACE-Southern analysis, probes/primers used, TATA box, poly-A site, mapped small RNAs, mapped FANTOM Paired-End Ditags (PET) sequences, which were obtained from the Ensembl website (www.ensembl.org), *Sox4ot1*, *Sox4ot2*, *Sox4*_sir3, untranslated regions, coding region and exons/introns.

The most important information within the Supplementary GenBank File is the mapped FANTOM Paired-End Ditags (PET) sequences. Twelve pairs of PET sequences were mapped to the locus indicating the presence of 6 different NATs. These NATs were named PET1-6 with 4 of them were successfully cloned and further analysed in Ling et al. [2]; PET2 (3214 bp), PET3 (1919 bp), PET5 (807 bp) and PET6 (1824 bp).

### 1.2 RNA Fluorescence *in situ* Hybridization (RNA FISH)

**Fig. 2:**
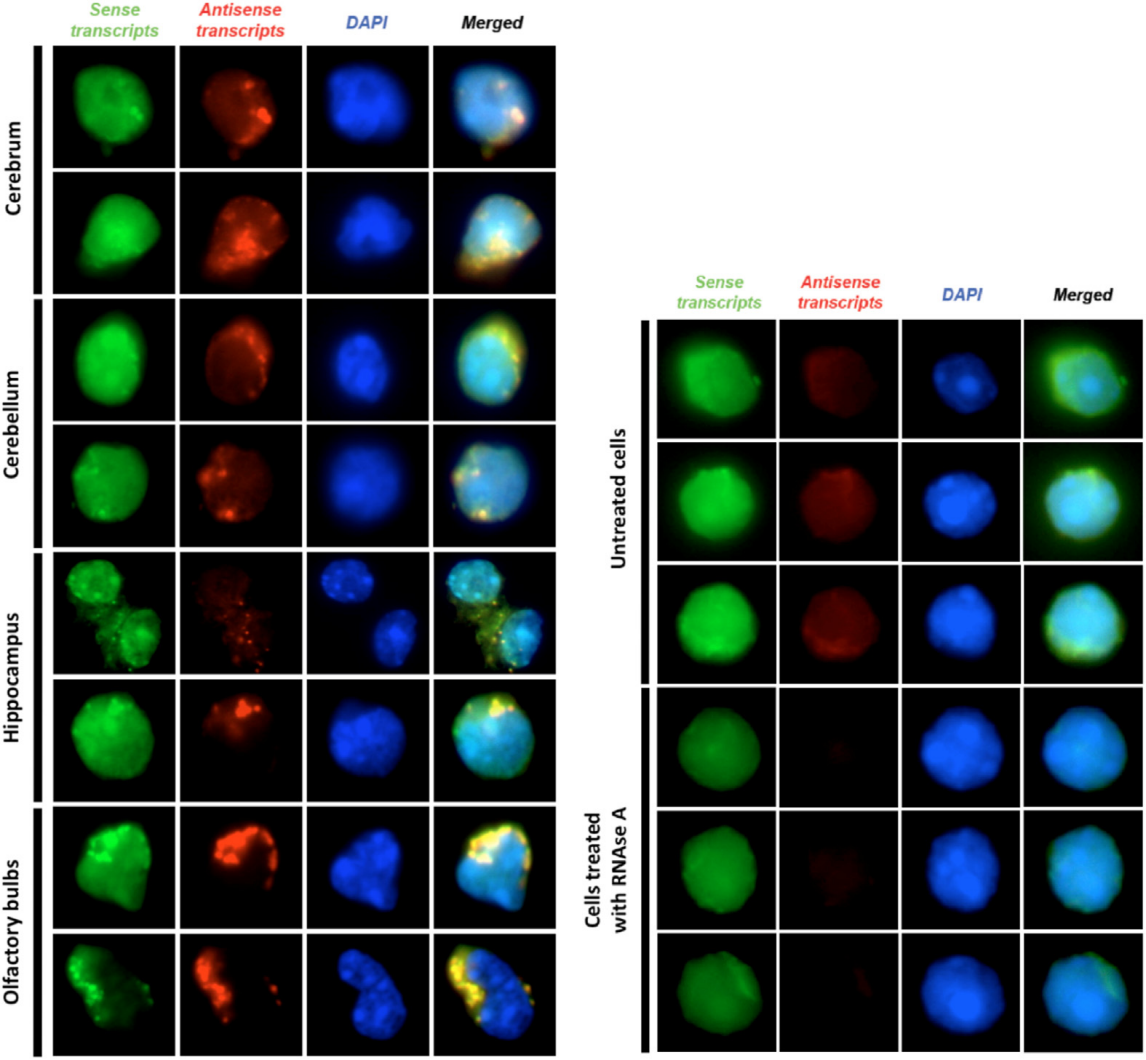
RNA FISH of *Sox4* and *Hmbs* sense and NATs. The type of transcripts analyzed is shown at the top of the figure and the origins of cells are shown to the left of the micrographs.

The data article also describes the results for RNA-FISH experiments performed on cells isolated from different regions of the mouse brain (Fig. 2). All cells presented here were treated with RNase A prior to hybridization step. From the micrographs, the signal of *Sox4* sense was generally diffused all over the cytoplasm whereas *Sox4* NATs were depicted as aggregates within the cytoplasm. Whenever *Sox4* NATs aggregates were observed, *Sox4* sense aggregates were found at the same loci within the cytoplasm.

**Fig. 3:**
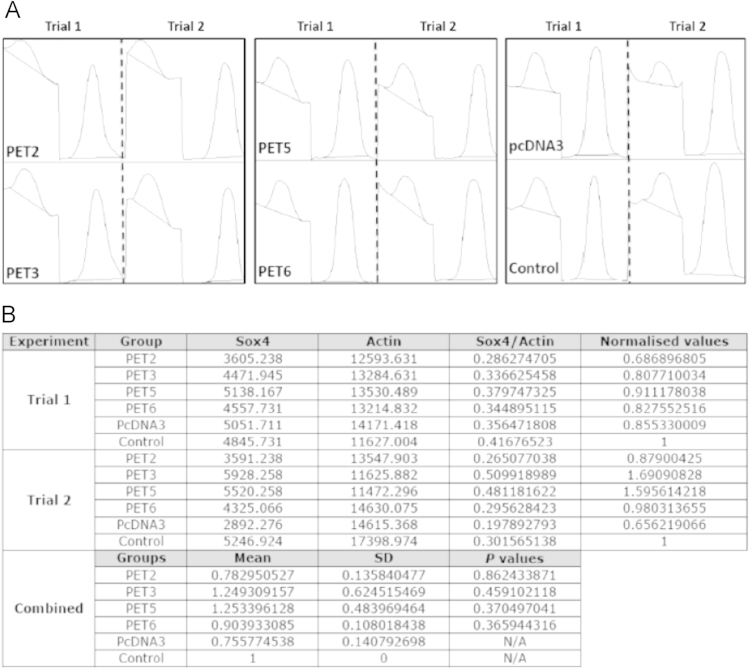
Pixelation analysis of bands generated from Western blotting experiments. (A) The area below each peak that was considered for pixel calculation. (B) Averages for calculated pixel values from two independent experiments were estimated and used for statistical analysis.

To control for RNase A treated FISH experiments for *Sox4*, RNA FISH was performed on cells obtained from P1.5 olfactory bulbs using probes against the *Hmbs* housekeeping gene (Fig. 2). To avoid biases, fluorescent micrographs were captured using a fixed exposure time for all channels. Exposure time was set to 500 ms for both FITC (sense transcripts) and TexasRed (antisense transcripts), and 10 ms for DAPI (nucleus) channels. Three untreated and 3 RNase A treated cells are shown in Fig. 3. Multiple images were obtained at the *Z*-axis and compiled into 8 different movie files, which have been compressed and provided as Supplementary Movies.

### 1.3 ImageJ pixelation analysis of bands generated from Western blotting experiments

We used ImageJ software (http://rsb.info.nih.gov/ij) to quantitatively estimate the intensity of bands in Western blotting experiments. Pixels from each band from two independent experiments were calculated by using a fixed rectangular selection approach (Fig. 3). Only area below each peak (defined as shoulder-to-shoulder cutoff) above the background noise was considered for pixel calculation. Total pixels of the Sox4 band from each group were then normalized against total pixels calculated from actin of the corresponding group. Similar steps were repeated for trial 2 of the experiment. Unpaired *T*-test (2-tailed) was used to compare PET/pcDNA3 and control groups for any significant differences but none of the *p*-values were lesser than 0.05.

### 1.4 Mapping of small RNA sequences at *Sox4* gene locus

**Fig. 4:**
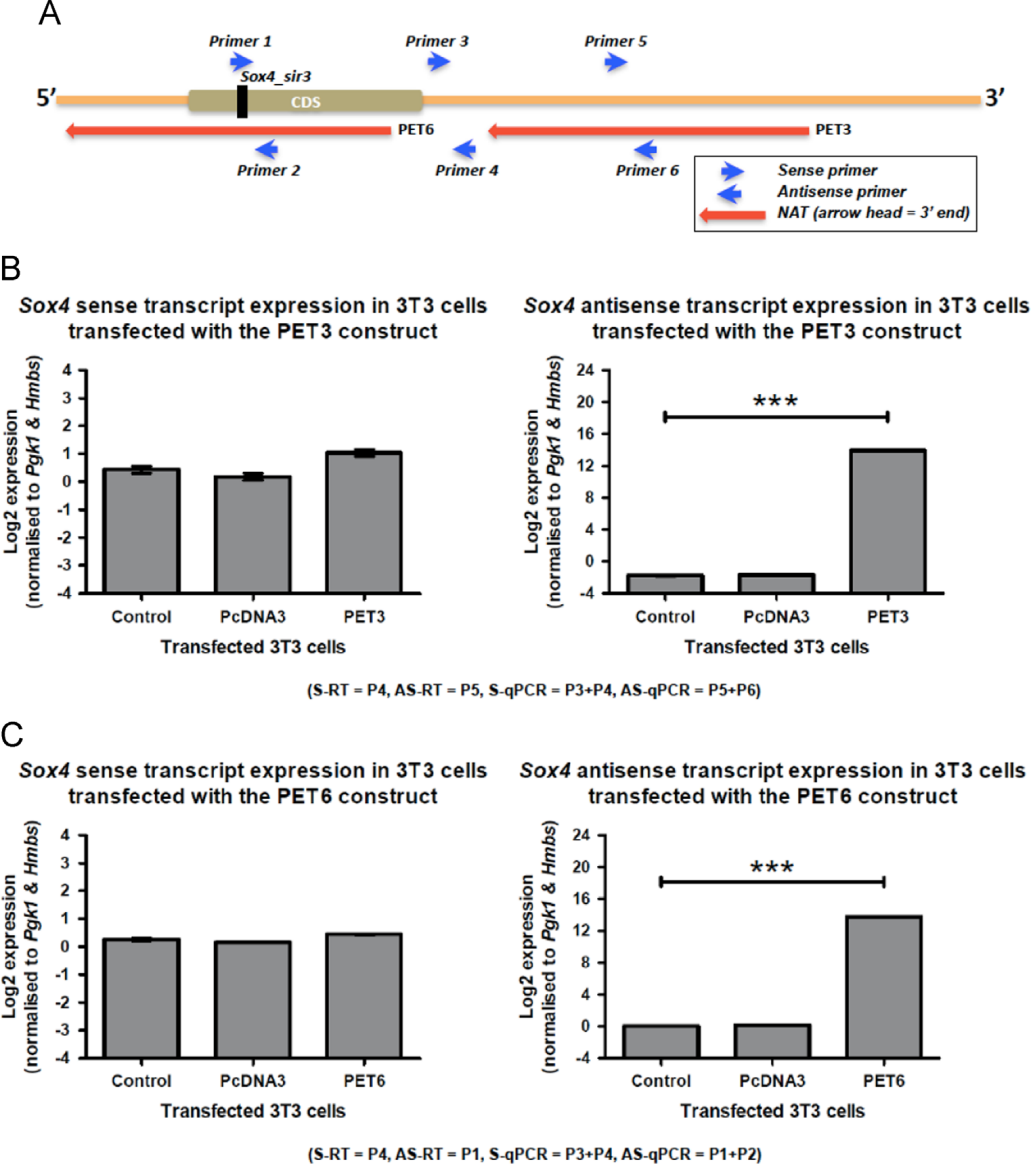
The strand specific RT-qPCR of Sox4 sense and antisense transcripts after PET3 and PET6 overexpression. (A) A schematic diagram represents the overlapping regions between the *Sox4* sense transcript, and the PET3 and PET6 NATs. *Sox4_sir3* and primers used (Primers 1–6) for RT-qPCR are also mapped. Normalised log2 expression level of *Sox4* sense (assessed by primers 3 and 4) and NATs in NIH 3T3 cells transfected with reagent only (control), pcDNA3-empty vector (pcDNA3) and individual pcDNA3-PET construct is illustrated in (B) for PET3 and (C) for PET6. For both (B) and (C), primers used during the sense- (S-RT) or antisense reverse-transcription (AS-RT) and sense- (S-qPCR) or antisense-quantitative PCR (AS-qPCR) are given in parentheses located below each graph. *N*=3 per group and asterisks denote significant level at *** *P* <0.001. Error bars denote the standard error of the mean.

To determine whether *Sox4* overlapping gene locus give rise to any small RNAs, we compared each *Sox4* gene sequence with ~3.7 million small RNA sequences generated from a mouse E15.5 whole brain using a massively parallel sequencing platform, the Illumina Genome Analyzer II (GSE22653) [4]. Only 7 small RNAs were matched and mapped to *Sox4* gene locus (Table 1). All the mapped sequences were mapped to the sense strand of the *Sox4* gene. The schematic diagram depicting the mapping of these small RNA at *Sox4* gene locus is shown in Fig. 4A.

### 1.5 Transfection analysis involving PET3 and PET6 NATs

Of all the mapped small RNAs, only *Sox4*_sir3 was determined as legitimate small RNA which originated from *Sox4* sense transcript. To determine whether *Sox4*_sir3 biogenesis may require the present of any *Sox4* NATs, we transfected NIH/3T3 cells with plasmids expressing PET3 (NAT that does not overlap the *Sox4*_sir3 origin site) and PET6 (NAT that overlaps *Sox4*_sir3 origin site). The overexpression of PET3 and PET6 both did not alter the level of sense transcript expression. As expected, the expression of the *Sox4* NAT at region overlapped by PET3 and PET6 were significantly upregulated (Fig. 4B and C).

### 1.6 Full-length sequencing of unspliced PET6 (*Sox4ot1*) and spliced PET6 (*Sox4ot2*)

**Fig. 5:**
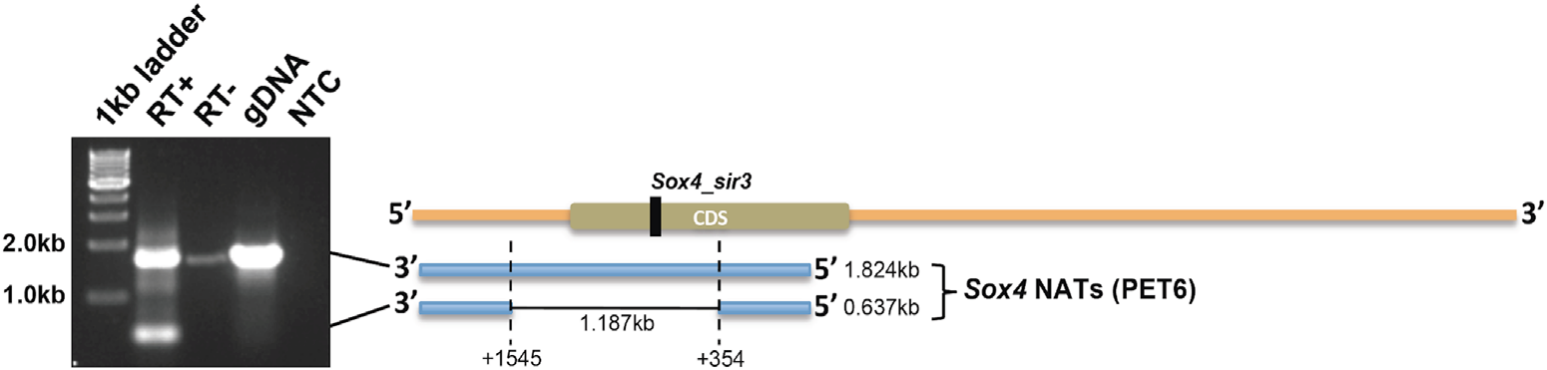
Sequencing of PET6 transcripts expressed in NIH/3T3-transfected cells. RT-PCR of PET6 NATs expressed in NIH 3T3-transfected cells revealed 2 transcript variants, which is schematically illustrated in the diagram next to the gel. RT+ denotes full RT-PCR reaction performed on the total RNA isolated from 3T3-transfected cells, RT− denotes a reaction without reverse transcriptase performed on the same sample during RT step (genomic DNA contamination control), gDNA denotes RT-PCR performed on ~100 ng mouse genomic DNA (positive control) and NTC denotes no template control.

**Fig. 6:**
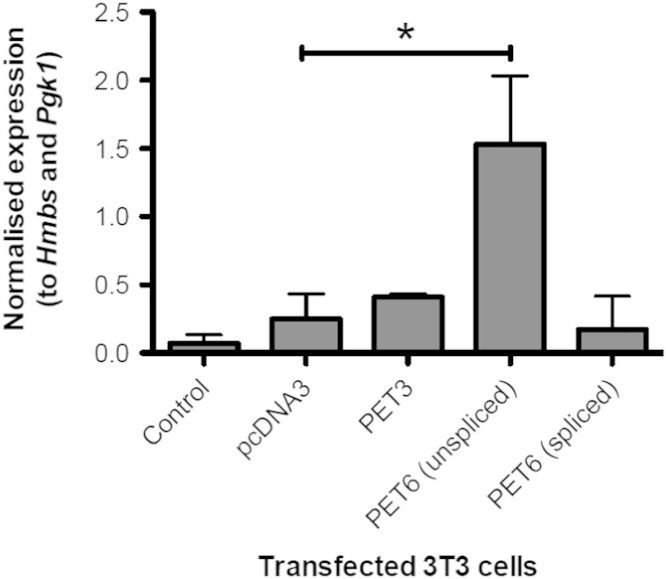
Overexpression analysis of PET6-(unspliced) and PET6-(spliced) in NIH/3T3 cells. NIH/3T3 cells were transfected with different constructs to determine the effect of spliced and unspliced variants of PET6 on the *Sox4*_sir3 expression level.

PET6 NATs were isolated from PET6 transfected NIH/3T3 cells. Amplifications were performed using the paired-end ditags sequences as primers (see Supplementary GenBank File). Amplicons were analysed using agarose gel electrophoresis to estimate the size of PET6. The analysis showed that there were 2 forms of PET6 NATs, one is unspliced and the other one is spliced (Fig. 5). Sanger DNA sequencing of purified amplicons were performed and the outcome confirmed both forms of PET6 sequence variants. Subsequent transfection analysis using both forms of PET6 variants showed that only the unspliced PET6 was involved in the induction of *Sox4*_sir3 small RNA (Fig. 6).

### 1.7 Scramble control for Locked Nucleic Acid (LNA)-in situ hybridization (ISH) analysis of *Sox4*_sir3

**Fig. 7:**
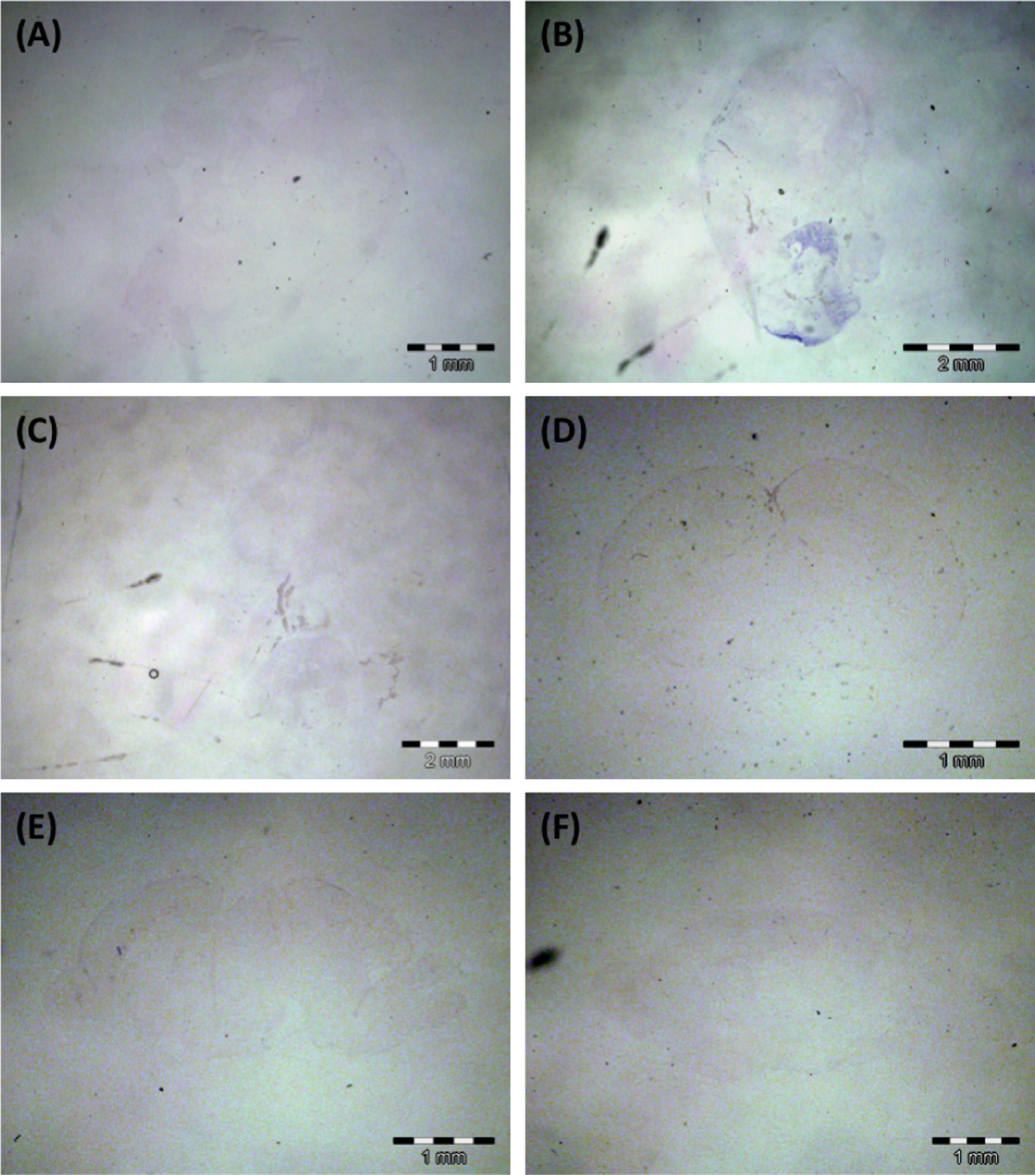
Scramble control for Locked Nucleic Acids – in situ hybridisation (LNA-ISH) analysis of *Sox4*_sir3 in whole mouse embryo and brain sections. (A) E11.5 whole embryo sagittal section, (B) E13.5 whole embryo sagittal section, (C) E15.5 whole embryo sagittal section, (D) E17.5 whole brain coronal section, (E) E17.5 whole brain sagittal section and (F) P1.5 whole brain sagittal section.

It is important that all *in situ* hybridization experiments are appropriately controlled to avoid misinterpretation of noisy signals. Locked Nucleic Acid (LNA)-*in situ* hybridization (ISH) for small RNA is usually controlled with a scramble probe, a mutated antisense probe or a sense probe. As the control for *Sox4*_sir3 LNA-ISH reported in Ling et al. [2], all corresponding serial whole embryo or brain sections were probed with the scramble probe (Exiqon) at the same temperature, washing stringency and colour development duration set for *Sox4*_sir3 probe (Fig. 7). The scramble control experiments showed low background colour development suggesting a successful LNA-ISH experiment.

### 1.8 RT-qPCR and statistical analysis

We adopted reverse-transcription quantitative polymerase chain reaction (RT-qPCR) to determine the relative levels for various Sox4 sense and NATs expression. All RT-qPCR data presented here were conforming to the criteria described elsewhere [1], [2], [4]. In all relative quantification analysis, One-way Analysis of Variance (ANOVA) was used to compare the expression levels among groups, brain tissues or mouse organs. The detail statistical analyses for this data and other data presented in [2] is provided in Supplementary Results.

## Figures and Tables

**Table 1:** Mapped small RNA sequences at the *Sox4* gene locus.

***ID***	***Sequence***	***nt***	***%GC***	***Mapping***	***Sox4 strand***	***Sox4 region***
*Sox4*_sir1	aggcggagagtagacggg	18	67	chr13:29043245-	sense	3׳UTR
*Sox4*_sir2	ccactggggttgtacgaa	18	56	chr13:29044007-	sense	CDS
*Sox4*_sir3	tcaaggacagcgacaagattccgt	24	50	chr13:29044567-	sense	CDS
*Sox4*_sir4	tcagggaaaggggtggggga	20	65	chr13:29045181-	sense	5׳UTR
*Sox4*_sir5	agacgatgtcgctttcctga	20	50	chr13:29045235-	sense	5׳UTR
*Sox4*_sir6	ggacttaggcgctagag	17	59	chr13:29045252-	sense	5׳UTR
*Sox4*_sir7	aggcgctagagacgatgt	18	56	chr13:29045246-	sense	5׳UTR
