# Supplementary material for: In depth analysis of the Sox4 gene locus that consists of sense and natural antisense transcripts
Source: Data Brief. 2016 Feb 17;7:282–90. doi: 10.1016/j.dib.2016.01.045 (PMC4773576; doi:10.1016/j.dib.2016.01.045)
Supplement: Supplementary file 1 — Supplementary material [file mmc1.zip › Supplementary Results.docx]

**Supplementary Results**

***Statistical analysis for RT-qPCR data***

***Reference:*** Figure 3A in [1]

***Statistical Methods:*** One way Analysis of Variance, with contrasts comparing *Sox4* sense expression in pcDNA3-empty, PET2, PET3, PET5 and PET6 to the blank control.

***Results:*** *Sox4* sense expression levels in pcDNA3-empty, PET3 and PET6 were not significantly different from the control. *Sox4* sense expression levels in PET2 and PET5 were both significantly different to the control (*P*<0.001 and <0.01 respectively). *Sox4* sense expression level in PET2 expression was 2.50 times the control, and the level in PET5 was 1.79 times the control. For each treatment n=2, and the standard error of the mean = 0.14.

| Transcripts | Log2 of normalized expression | | | | | |
| --- | --- | --- | --- | --- | --- | --- |
|  | Control | pcDNA3 | PET2 | PET3 | PET5 | PET6 |
| Mean Sense | 2.32 | 2.28 | 3.64 | 2.03 | 3.16 | 1.88 |
| *P* (Not different to control) |  | 0.84 | <0.001 | 0.19 | 0.005 | 0.07 |
| Significance Level |  | N.S | *** | N.S | ** | N.S |

***Statistical Methods:*** One way Analysis of Variance, with contrasts comparing Sox4 antisense expression in pcDNA, PET2 and PET3 to the control.

***Results:*** *Sox4* antisense expression level in pcDNA3-empty was not significantly different from the control. *Sox4* antisense expression levels in PET2 and PET3 were both significantly different from the control (*P*<0.001 in both cases) with 21 and 14 times the control, respectively. For each treatment n=2, and the standard error of the mean = 0.20.

| Transcripts | Log2 of normalized expression | | | |
| --- | --- | --- | --- | --- |
|  | Control | pcDNA3 | PET2 | PET3 |
| Mean Antisense | -0.59 | -0.81 | 3.81 | 3.17 |
| *P* (Not different to control) |  | 0.50 | <0.001 | <0.001 |
| Significance Level |  | N.S. | *** | *** |

***Statistical Methods:*** One way Analysis of Variance, with contrasts comparing *Sox4* antisense expression levels in pcDNA3-empty and PET5 to the control.

***Results:*** *Sox4* antisense expression level in pcDNA3-empty was not significantly different from the control. *Sox4* antisense expression level in PET5 was significantly different from the control (*P*<0.01) with 28 times the control. For each treatment n=2, and the standard error of the mean = 0.32.

| Transcripts | Log2 of normalized expression | | |
| --- | --- | --- | --- |
|  | Control | pcDNA3 | PET5 |
| Mean Antisense | -2.34 | -2.50 | 2.48 |
| *P* (Not different to control) |  | 0.74 | 0.002 |
| Significance Level |  | N.S. | ** |

***Statistical Methods:*** One way Analysis of Variance, with contrasts comparing *Sox4* antisense expression levels in pcDNA3-empty and PET6 to the control.

***Results:*** *Sox4* antisense expression level in pcDNA3-empty was not significantly different from the control. *Sox4* antisense expression level in PET6 was significantly different to the control (*P*<0.001) with 376 times the control. For each treatment n=2, and the standard error of the mean = 0.067.

| Transcripts | Log2 of normalized expression | | |
| --- | --- | --- | --- |
|  | Control | pcDNA3 | PET6 |
| Mean Antisense | -0.426 | -0.445 | 8.130 |
| *P* (Not different to control) |  | 0.85 | <0.001 |
| Significance Level |  | N.S. | *** |

***Reference:*** Figure 4D in [1]

***Statistical Methods:*** One way Analysis of Variance for the Dicer1 effect in both the sense and antisense transcripts.

***Results:*** There was no significant difference in expression of *Sox4* sense transcripts between the mES cells with Dicer1 c/- and Dicer1 -/- (*P*=0.32). There was a significant difference in expression of *Sox4* antisense transcripts between the mES cells with Dicer1 c/- and Dicer1 -/- (*P*=0.004). The mES cells with Dicer1 c/- had 3.6 times the Sox4 antisense expression of mES cells with Dicer1 -/-. For each cell type, n=3.

| Transcript | mES cells with conditional Dicer1 allele | | SEM | *P* (No Dicer1 Effect) | Significance Level |
| --- | --- | --- | --- | --- | --- |
|  | c/- | -/- |  |  |  |
| Sense | -9.20 | -9.66 | 0.29 | 0.32 | N.S. |
| Antisense | -12.07 | -13.91 | 0.21 | 0.004 | ** |

***Statistical Methods:*** One way Analysis of Variance of Dicer1 expression between Dicer 1 c/- and -/- groups.

***Results:*** The expression level of Dicer1 in mES cells with Dicer1 c/- was significantly (*P*<0.001) different from mES cells with Dicer1 -/-. Dicer1 expression in mES cells with Dicer1 c/- expression was almost 10 times the mES cells with Dicer1 -/-. For both type of cells, n=3 and the standard error of the mean was 0.16.

| mES cells | Mean (log2 normalized expression) |
| --- | --- |
| Dicer1 c/- | 1.51 |
| Dicer1 -/- | -1.74 |

***Statistical Methods:*** One way Analysis of Variance of Sox4_sir3 expression between Dicer 1 c/- and -/- groups.

***Results:*** The *Sox4_sir3* expression level of mES cells with Dicer1 c/- was significantly (*P*=0.03) different from the mES cells with Dicer1 -/-. *Sox4_sir3* expression level of mES cells with Dicer1 c/- was almost 24 times the mES cells with Dicer1 -/-. For both cell types, n=3 and the standard error of the mean was 0.99.

| mES cells | Mean (log2 normalized expression) |
| --- | --- |
| Dicer c/- | -6.78 |
| Dicer -/- | -11.36 |

***Reference:*** Figure 5 in [1]

***Statistical Methods:*** One way Analysis of Variance, with each treatment compared to the control (contrasts).

***Results:*** For Figure 5A: There was no significant difference in the *Sox4_sir3* expression in either pcDNA3-empty or PET3 and the control (*P*=0.86 and 0.24 respectively). There was a significant difference in *Sox4_sir3* expression between PET6 and the control (*P*<0.001). In PET6, *Sox4_sir3* was expressed approximately 70 times the expression in the control. For each group, n=3, and the standard error of the mean = 0.51.

For Figure 5B: The pcDNA3-empty, PET3 and PET6 did not have significantly different Dicer1 expression levels from the control (*P*=0.36, 0.32 and 0.56, respectively). For each group, n=3, and the standard error of the mean = 0.058.

| Group | For Figure 5A | | | For Figure 5B | | |
| --- | --- | --- | --- | --- | --- | --- |
|  | Mean | *P* (Not different from control) | Significance Level | Mean | *P* (Not different from control) | Significance Level |
| Control | -8.94 |  |  | 0.194 |  |  |
| pcDNA | -8.77 | 0.86 | N.S. | 0.096 | 0.36 | N.S. |
| PET3 | -10.07 | 0.24 | N.S. | 0.088 | 0.32 | N.S. |
| PET6 | -2.79 | <0.001 | *** | 0.134 | 0.56 | N.S. |

***Reference:*** Figure 7A in [1]

***Statistical Methods:*** One way Analysis of Variance analysis to determine if there are differences in *Sox4_sir3* expression levels among the different developmental stages of the brain.

***Results:*** There was a significant difference among the *Sox4_sir3* expression levels (*P*<0.01). The expression levels fell into 3 significantly different groups: P1.5 had higher *Sox4_sir3* expression levels than all the other ages; and E15.5, E17.5 and P150 had significantly lower *Sox4_sir3* expression levels than the other ages. The means and standard errors for the brain expression at each age are given in the table below. There were 2 replicates for each age, the standard error for each mean was 0.32, and LSD = 1.10.

| Age | Mean |
| --- | --- |
| Embryonic Day 11.5 | -0.06 |
| Embryonic Day 13.5 | -0.22 |
| Embryonic Day 15.5 | -1.36 |
| Embryonic Day 17.5 | -2.00 |
| Postnatal Day 1.5 | 1.14 |
| Postnatal Day 150 | -1.49 |

***Reference***: Figure 7B in [1]

***Statistical methods:*** One way Analysis of Variance analysis to determine if there are differences in *Sox4_sir3* expression levels among the different brain regions in adult mice.

***Results:*** There was a significant difference in the expression levels of *Sox4_sir3* among the brain organs (P=0.03). The cerebellum had significantly lower expression of *Sox4_sir3* than the cerebrum, hippocampus, olfactory bulb and medulla. The mean log2 normalized expression level is given in the following table for each brain region. For each brain region there are 2 replicates, the standard error is 0.57, and LSD = 1.98.

| **Brain region** | **Mean** |
| --- | --- |
| Cerebellum | -3.02 |
| Olfactory bulb | 0.45 |
| Cerebrum | -0.57 |
| Medulla | 0.51 |
| Thalamus | -1.16 |
| Hippocampus | -0.33 |

***Reference:*** Figure 7C in [1]

***Statistical methods:*** One way Analysis of Variance to determine if there are differences in *Sox4_sir3* expression levels among the different organs in P150 adult mice.

***Results:*** There was no significant difference in the *Sox4_sir3* expression levels among the organs (P<0.33). The mean log2 normalized expression level is given in the following table for each organ. Group A organs have three replicates, and their standard error is 1.42, whereas group B organs have two replicates, and their standard error is 1.74.

| **Organ** | **Mean** | **Group** |
| --- | --- | --- |
| Spleen | -2.16 | A |
| Stomach | -3.35 | A |
| Liver | -5.78 | B |
| Skeletal muscle | -1.88 | A |
| Skin | -0.52 | A |
| Ovary | -1.79 | B |
| Testes | -2.33 | B |
| Brain | -1.49 | B |
| Kidney | 0.02 | B |
| Small intestine | -3.57 | B |
| Heart | 0.53 | B |
| Large intestine | -1.98 | B |
| Thymus | -1.55 | B |
| Pancreas | 1.94 | B |

***Reference:*** Figure 4 in [2]

***Statistical Methods:*** One way Analysis of Variance, with contrasts comparing *Sox4* transcript expression levels in pcDNA3-empty and PET3 with the control.

***Results:*** For *Sox4* sense transcript: the expression levels in neither the pcDNA3-empty nor PET3 were significantly different from the control (*P*= 0.53 and 0.17 respectively). For each group, n=3, and the standard error of the mean = 0.22.

| Sox4 sense transcript levels | Log2 of normalized expression | | |
| --- | --- | --- | --- |
|  | Control | pcDNA3 | PET3 |
| Mean Sense | 0.44 | 0.18 | 1.03 |
| *P* (Not different to control) |  | 0.53 | 0.17 |
| Significance Level |  | N.S. | N.S. |

For *Sox4* antisense transcript: the expression level of the pcDNA3-empty was not significantly different from the control (*P*=0.78). The expression level of PET3 was significantly different from the control (*P*<0.001). PET3 expression was over 52,000 times the expression of the control. For each group, n=3, and the standard error of the mean = 0.19.

| Sox4 antisense transcript levels | Log2 of normalized expression | | |
| --- | --- | --- | --- |
|  | Control | pcDNA3 | PET3 |
| Mean Antisense | -1.76 | -1.67 | 13.91 |
| *P* (Not different to control) |  | 0.78 | <0.001 |
| Significance Level |  | N.S. | *** |

***Statistical Methods:*** One way Analysis of Variance, with contrasts comparing Sox4 transcript expression levels in pcDNA3-empty and PET6 to the control.

***Results:*** For *Sox4* sense transcript: The expression levels of neither the pcDNA3-empty nor the PET6 were significantly different from the control (*P*=0.34 and 0.11 respectively). For each group, n=3, and the standard error of the mean = 0.055.

| Sox4 sense transcript levels | Log2 of normalized expression | | |
| --- | --- | --- | --- |
|  | Control | pcDNA3 | PET6 |
| Mean Sense | 0.263 | 0.165 | 0.444 |
| *P* (Not different to control) |  | 0.34 | 0.11 |
| Significance Level |  | N.S. | N.S. |

For *Sox4* antisense transcript: The expression level of the pcDNA3-empty was not significantly different from the control (*P*=0.74). The expression level of PET6 was significantly different from the control (*P*<0.001). PET6 expression was over 13,000 times the expression level of the control. For each group, n=3, and the standard error of the mean = 0.14.

| Sox4 antisense transcript levels | Log2 of normalized expression | | |
| --- | --- | --- | --- |
|  | Control | pcDNA3 | PET6 |
| Mean Antisense | 0.08 | 0.17 | 13.76 |
| *P* (Not different to control) |  | 0.74 | <0.001 |
| Significance Level |  | N.S. | *** |

**References:**

[1] K.H. Ling, P.J. Brautigan, S. Moore, R. Fraser, P.-S. Cheah, J.M. Raison, et al., Derivation of an endogenous small RNA from double-stranded Sox4 sense and natural antisense transcripts in the mouse brain, Genomics, 10.1016/j.ygeno.2016.01.006.

[2] K.-H. Ling, P.J. Brautigan, S. Moore, R. Fraser, M.P.-Y. Leong, J.-W. Leong, et al., In depth analysis of the Sox4 gene locus that consist of sense and natural antisense transcripts, Data in Brief. (submitted).
